# Population Physiology: Leveraging Electronic Health Record Data to Understand Human Endocrine Dynamics

**DOI:** 10.1371/journal.pone.0048058

**Published:** 2012-12-14

**Authors:** D. J. Albers, George Hripcsak, Michael Schmidt

**Affiliations:** 1 Department of Biomedical Informatics, Columbia University, New York, New York, United States of America; 2 Department of Biomedical Informatics, Columbia University, New York, New York, United States of America; 3 Department of Neurology, Columbia University, New York, New York, United States of America; University of Vermont, United States of America

## Abstract

Studying physiology and pathophysiology over a broad population for long periods of time is difficult primarily because collecting human physiologic data can be intrusive, dangerous, and expensive. One solution is to use data that have been collected for a different purpose. Electronic health record (EHR) data promise to support the development and testing of mechanistic physiologic models on diverse populations and allow correlation with clinical outcomes, but limitations in the data have thus far thwarted such use. For example, using uncontrolled population-scale EHR data to verify the outcome of time dependent behavior of mechanistic, constructive models can be difficult because: (i) aggregation of the population can obscure or generate a signal, (ii) there is often no control population with a well understood health state, and (iii) diversity in how the population is measured can make the data difficult to fit into conventional analysis techniques. This paper shows that it is possible to use EHR data to test a physiological model for a population and over long time scales. Specifically, a methodology is developed and demonstrated for testing a mechanistic, time-dependent, physiological model of serum glucose dynamics with uncontrolled, population-scale, physiological patient data extracted from an EHR repository. It is shown that there is *no observable* daily variation the normalized mean glucose for any EHR subpopulations. In contrast, a derived value, daily variation in *nonlinear correlation* quantified by the time-delayed mutual information (TDMI), *did reveal* the intuitively expected diurnal variation in glucose levels amongst a random population of humans. Moreover, in a population of continuously (tube) fed patients, there was no observable TDMI-based diurnal signal. These TDMI-based signals, via a glucose insulin model, were then connected with human feeding patterns. In particular, a constructive physiological model was shown to correctly predict the difference between the general uncontrolled population and a subpopulation whose feeding was controlled.

## Introduction

Human physiology, as a science, aims to understand the mechanical, physical, and biochemical functions of humans; moreover, because human dynamics transpire both on multiple spatial scales, ranging from molecular (e.g., genetics), to cell (e.g., metabolism), to organ (e.g., the heart [1]), to collections of organs (e.g., the circulatory system) and on multiple time scales ranging from fractions of a second to decades, it is likely that complete models of human functioning will consist of highly complex models whose scales interact in complex ways (e.g., via nonlinear resonance) [2]. In this context, *population physiology* aims to understand medium to long time scales of human physiology and pathophysiology where *a population of humans* is required to construct or discover a signal (metaphorically, population physiology is to physiology as climatology is to weather). Moreover, once a signal is constructed, the goal is to use this signal to understand human dynamics by both understanding the sources of the signals and then use that information to stratify the population into meaningful classes (e.g., phenotypes) according to the different signals. Consequently, population physiology, as we conceive it, has two broad features: data analysis consisting of the construction and analysis of population scale physiological signals, and the mechanistic modeling that can explain and rationalize those signals. The hope is that, through the use of EHR data, physiology can eventually be used by clinicians in the same way that physics is used by engineers. Thus, here we will employ diverse populations in an attempt to verify that an EHR-data-derived signal can be used to resolve *first-order* physiologic dynamics.

The mathematical modeling of physiological systems on the cellular and organ scales has a long history (cf., [3] and [4] for a wonderful introduction), while the modeling of larger scale organ structures is just beginning [5]. Fundamental to mathematical modeling of physiology is a concrete connection to real data; as is the case for other basic sciences, mathematical physiological modeling is always tested against physiological data collected in rigorously controlled circumstances. Nevertheless, there are at least two elements missing from modern physiological analysis, analysis over large populations and analysis over long time periods. The former is important because human beings have diverse reactions to different inputs (e.g., drugs, foods, etc.), and those differences have their roots in physiology. The latter is important because many differences amongst human reactions to input occur on a slow time-scale; for instance, some smokers develop cancer while others do not. The problem with using the classical physiology framework with its rigorously controlled conditions to study a large population over a long time period is that it is too expensive, intrusive, and dangerous to collect physiologic data for a large population over a long time period. Thus, it is likely that the lack of availability of population scale, long term data is the primary reason why wide-population, long term, physiologic studies to not exist.

With the advancement of electronic health record (EHR) repositories, the “lack of data” problem will be replaced with data analysis and data mining problems. Electronic health records hold data for large, diverse populations, and they cover periods of decades [6] [7] [8]. Nevertheless, despite years of work, the methods needed to exploit EHR data remain in their infancy. A necessary realization for using EHR data is recognizing that the EHR represents a natural system in its own right. In particular, EHR data not only represents the physiology of the diverse population being cared for, but also the following: healthcare measurement dynamics (e.g., individual hospital protocols); the local environment (e.g., exposure to pollutants); local customs (e.g., willingness to seek medical attention); and any other features of the environment in which the data are collected. To see some of the difficulties and potential associated with the analysis of EHR data, consider four notably relevant examples: Sagreiya and Altman [9] demonstrated the limitations of using general population EHR data for estimating drug dosages; Hripcsak *et al.* [10] showed the difficulties with using general EHR data for classification of disease (i.e., community-acquired pneumonia); Karsh *et al.* [8] outlined various factors that will constrain EHR data; and Higgins and Mehadevan [11] demonstrated that relevant, predictive, phenomenological master equations of physiological functioning (concentrations of red blood cells) can be generated using data that *could* exist in an EHR repository (note that in Higgins and Mehadevan [11] the terminology *population dynamics* refers to a population of red blood cells not humans) and that, if integrated into a EHR infrastructure, would help with early prevention of disease (i.e., anemia). Advancing such methods is a step-wise process, and here we present what we believe is an important early step: showing that it is feasible to use EHR data in conjunction with a constructive physiological model — specifically, that we can test a physiologic model with an EHR data-derived signal.

To study how EHR data can be used in conjunction with a physiological model, we consider the relatively simple problem of glucose variation because it is easy to present and understand, it has relevant, well understood models, and we know what the answer should be. Specifically, we leverage the following tools or data sets: **(i)** a subpopulation of patients with at least two glucose measurements from an EHR that includes all inpatients and outpatients seen at an academic medical center over 20 years; **(ii)** two well sampled patients from the same previously mentioned EHR; **(iii)** a set of particularly sick, continuously-fed (via a feeding tube), immobile, comatose patients taken from the neural intensive care unit (NICU) portion of the previously mentioned EHR; **(iv)** a relatively simple mechanistic glucose-insulin model with various different feeding patterns; and **(v)**, the time-delay mutual information (TDMI) which quantifies *nonlinear correlation* between *ensembles* of measurements separated by a given amount of time.

Along with demonstrating that EHR data can be used to test physiologic models for populations over long time periods, we also discover that while human glucose levels are highly aperiodic, there is nevertheless a stable, long term diurnal structure in the *nonlinear correlation* between glucose values separated in time in healthy, random humans. Moreover, while it is likely that many features contribute to the observed diurnal cycle in correlated glucose, only *two* interacting time scales are required to reproduce the observed diurnal signal — a “statistically periodic” feeding pattern that exists on the scale of weeks and the organ level dynamics that exists on the order of minutes. Less broadly, we find that: **(i)** to first order statistical moment (e.g., the mean), daily variation in the TDMI is a function of feeding alone—no diversity in other parameters that determine glucose/insulin regulation are required; **(ii)** that glucose regulation acts like a control system on a fast time scale (order of minutes) in contrast to kidney function which behaves like a filtering system [12]; **(iii)** a diurnal signal in a derived value, nonlinear correlation (TDMI), that can be used to distinguish different populations; and **(iv)** it is possible to circumvent inter-patient variability though aggregating populations, but one must be very careful interpreting the results [13].

### Outline

We begin with a materials and methods section that has three distinct components. In subsection 0.3 we discuss endocrine physiology and introduce the mechanic model we use in this paper. We then introduce electronic health record data in general and the data we use in particular in subsection 0.4. The *materials and methods* section concludes with a discussion of the nonlinear time series analysis techniques we use (subsection 0.5). We then work through the results (section 0.5) and discussion (section 0.9) sections.

## Materials and Methods

### 0.1 Ethics statement

This work was approved by the Columbia University Institutional Review Board. Informed consent was waived by the Institutional Review Board for this retrospective research.

### 0.2 Data assess statement

Unfortunately, the data for this study cannot be made publically available because the detail and complexity of the data put it at risk for re-identification. Similar data are publically available from the PHYSIONET and MIMIC data repositories.

### 0.3 Glucose-Insulin physiology

#### 0.3.1 Background: endocrine dynamics

Begin by noting that a complete physiological understanding of the endocrine system, or even the glucose/insulin cycle, has not yet been achieved. For instance, how insulin reacts at the plasma membrane of insulin sensitive cells is still poorly understood (for other examples, cf., [14] [15]). With respect to diurnal cycles in glucose/insulin dynamics, the following effects have been observed: in *fasting humans*, there are wake-sleep cycle based effects on pancreatic enzyme secretions [16]; physical activity has an effect on insulin secretion [17]; and in rats there appears to be an endogenous circadian oscillator (internal clock) located within the pancreatic islets [18]. Most importantly, it is well understood that nutrition intake is the primary first order driver of the glucose-insulin cycle [17] (hence the need to use fasting humans as a control to isolate the more sensitive glucose-insulin effects). All of these studies were carried out under the classical physiology framework. Moreover, to resolve many of the previously listed signals required rigorous control of the measured individuals—most EHR data will never meet these standards. But, the noted contrast between classical physiology data and EHR data helps clarify one of the goals of this paper: we are not trying to discover an ultra-sensitive, controlled, physiological effect that is resolvable over a short time period; rather, we are trying to discover what can be resolved with EHR data. Specifically, we are trying to discover gross, long term, population-wide effects that have the potential to help stratify populations into observably different types — types that can eventually be linked to different health states. Moreover, because the individuals within the EHR have observably differing health states that do not require ultra-fine resolution to observe, the hope is that we will be able to eventually use EHR data to discover and categorize different, long term, physiologic macrostates. This is the justification for not choosing the most complicated glucose/insulin model. While the model we utilize parameterizes away many of these higher-order effects, it remains driven by nutrition, the source of the first order, elementary glucose/insulin dynamics we are trying to verify.

#### 0.3.2 First principles model of glucose-insulin physiology

The first principles, constructive, mechanistic glucose-insulin we use is presented in Sturis *et al.* [19] which consists of six ordinary differential equations (ODEs), specifically:
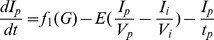
(1)

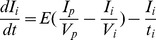
(2)

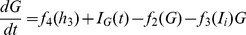
(3)and a three stage linear filter:
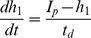
(4)

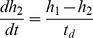
(5)

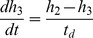
(6)where the state variables correspond to: 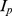, plasma insulin; 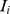, remote insulin; 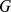, glucose; and 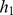, 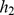 and 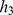 which correspond to three parameterized delay processes. The *major* parameters include: (i) 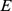, a rate constant for exchange of insulin between the plasma and remote compartments; (ii) 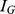, the exogenous (externally driven) glucose delivery rate; 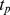, the time constant for plasma insulin degradation; (iii) 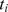, the time constant for the remote insulin degradation; (iv) 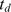, the delay time between plasma insulin and glucose production; (v) 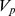, the volume of insulin distribution in the plasma; (vi) 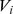, the volume of the remote insulin compartment; (vii) 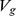, the volume of the glucose space; (viii) 
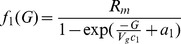
, insulin secretion; (ix) 
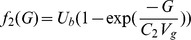
, insulin-independent glucose utilization; (x) 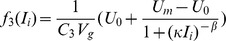, insulin-dependent glucose utilization (
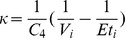
); and (xi) 
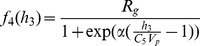
, insulin-dependent glucose utilization. Note that a full list of the parameters in this model, as well the model parameter settings used in this paper, can be found in table 1; moreover, Keener and Sneyd [4] provides a nice discussion of this particular model. With the exception of the exogenous glucose delivery rates, which we will discuss shortly, we utilize all the standard parameter settings used in Sturis *et al.* [19]. Finally, there do exist more complex, higher order glucose/insulin metabolism models [20], but because the point was to choose the simplest system of ODEs that can be used to represent the data-driven signal, we chose this rather standard model.

**Table 1:** Full list of parameters for the glucose/insulin model [19] used in this paper.

Glucose model parameters		
Parameter	nominal value	meaning
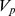	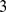 l	plasma volume
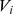	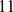 l	insulin volume
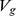	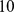 l	glucose space
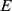	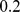 l min^−1^	exchange rate for insulin between remote and plasma compartments
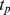	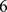 min	time constant for plasma insulin degradation (via kidney and liver filtering)
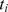	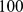 min	time constant for remote insulin degradation
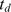	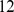 min	delay between plasma insulin and glucose production
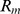	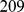 mU min^−1^	linear constant affecting insulin secretion
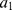	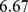	exponential constant affecting insulin secretion
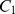	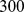 mg l^−1^	exponential constant affecting insulin secretion
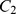	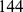 mg l^−1^	exponential constant affecting IIGU
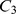	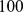 mg l^−1^	linear constant affecting IDGU
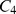	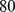 mU l^−1^	factor affecting IDGU
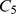	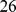 mU l^−1^	exponential constant affecting IDGU
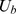	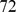 mg min^−1^	linear constant effacing IIGU
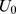	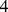 mg min^−1^	linear constant affecting IDGU
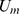	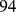 mg min^−1^	linear constant affecting IDGU
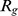	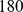 mg min^−1^	linear constant affecting IDGU
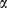	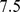	exponential constant affecting IDGU
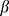	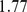	*exponent* affecting IDGU

Note that these are the model parameters we us in this paper. Note the following abbreviations: insulin independent glucose utilization (IIGU) and insulin dependent glucose utilization (IDGU).

The only part of the model we vary is the external driving, or the *exogenous glucose delivery rate*, 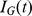; specifically, we consider five different feeding patterns. The first feeding pattern we consider is a population that is fed continuously and where each member of the population is fed at a different rate. This feeding pattern forms a baseline for other continuous and periodically fed populations and is denoted by the feeding function 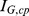. The data sets generated with this feeding structure include 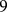 days of data collected by the minute. The second feeding pattern is identical to the first with the exception that 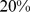 of the 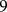 days of data have *randomly selected* four hour gaps where no food is administered. This feeding pattern is meant to simulate an intensive care unit population and is denoted by the feeding function 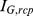. Both of these feeding patterns are, in a sense, pathophysiologic. The other three feeding patterns are based on simulated meals. To construct mealtime feeding structure, begin by defining the set of meal times, specified by the set 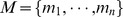, where the 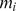
's represent times over a 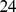-hour interval, and 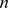 is the number of meal times within a 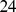-hour period. Next define the exogenous glucose delivery rate at the current time, 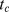, as:
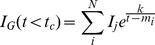
(7)where 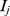 is the peak rate of delivery of glucose for a given individual 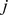 at time 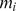, 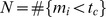 represents the total number of meals that have passed by time 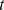, and 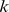 is the decay constant (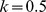). The decay constant is set such that the meal persists over about two hours, a time that is considered realistic [19]. Next, relative to the 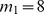, 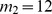, and 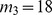, define the following three feeding patterns: *periodic individual*, 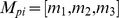; *noisy individual*, 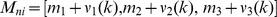 where 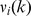 is a uniform random variable on the interval 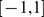 and 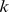 represents an integer day (implying that 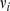 changes every day); and *random individual*, 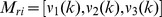 where 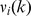 is a random (non-repeated) integer on the interval 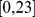 and 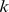 is again an integer day (implying that 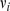 changes every day). Based on these meal structures we define five feeding patterns, *continuously fed population* (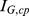), *continuously fed population with random gaps* (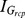), a periodically fed individual (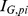), a noisy-periodic individual (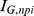), and a random individual (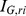), defined formally as:
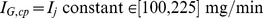
(8)

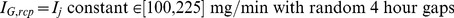
(9)

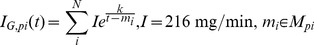
(10)

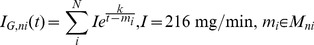
(11)

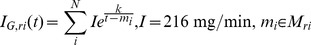
(12)These five different driving mechanisms reflect what we believe to be a relatively minimalistic amount of variation within the glucose/insulin model parameter and function space.

#### 0.3.3 Endocrine dynamics as a control system

To interpret the results, it will help to abstract the physical mechanisms to a control system. In particular, the regulation of glucose can be thought of as an intra-body feedback control system where the body *has a goal of maintaining a constant concentration of glucose* and attempts to achieve this goal via various physiological mechanisms [21]. Broadly, when glucose levels are high, insulin is released by the pancreas leading to glucose being stored in the liver faster than it is released *and* the rate at which glucose is metabolized by the body is increased. Similarly, when glucose levels are low, glucagon is released by the pancreas, allowing for an increase in the rate glucose is released from the liver as well as a decrease in the rate glucose is metabolized by the body. This contrasts with, for example, the kidneys and their relation with creatinine, which can be grossly thought of as a filtering system instead of a control system aiming at maintaining a particular level of glucose. (Note, there are *parts* of the kidney that do behave as a control system [22]). It is worth mentioning that the above description of the endocrine system is greatly simplified, (for a more detailed view, cf. [23] [24]).

### 0.4 Data composition

We consider the time series of glucose measurements of two real populations of humans extracted from the Columbia University Medical Center (CUMC) EHR: **(i)** the time series of glucose measurements extracted from an EHR for all inpatients and outpatients over 20 years (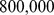 patients with roughly 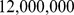 glucose measurements); (ii) the time series of glucose measurements for a small subset of patients (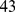 in total) seen in the NICU who are continuously fed, immobile, and comatose—note that this cohort of patients is represented by between 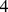 and 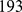 measurements taken on the order of minutes to hours (many patients have approximately a weeks' worth of hourly measurements). Finally, it is important to note that glucose is measured in many contexts, many of which include situations where glucose is not the primary target (e.g., the CHEM-7 metabolic panel, which includes glucose, is taken in many situations where glucose is not primary chemical of interest).

The data sets we chose are of varying size over populations, numbers of points, and time periods. Nevertheless, the population size is not explicitly important. Rather, it is the number of data points used to compute the given quantities that are of explicit importance. Specifically, the number of points are important because the errors or estimation biases of computable quantities (e.g., averages, TDMI, etc.) depend on the number of points, (cf. Albers and Hripscak [25] for a description for TDMI). Because EHR data are special in that it is not collected in a controlled environment, the EHR represents a population whose individuals are both diverse and measured diversely. Often only a small percentage of the population has the required characteristics for computation. Similarly, sometimes many sparsely measured individuals can be aggregated such that the aggregated population can accurately represent a single well-measured individual. Because of this, 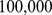 sparsely measured patients can essentially represent 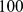 well measured patients. In all cases, the numbers of patients we have selected are arbitrary numbers that are large enough to compute trustable quantities.

#### 0.4.1 EHR population

The first data set, the EHR patients, is not filtered or carefully selected in anyway; we chose to use the entire EHR population for five reasons. *First*, we want to demonstrate the generality of the first order effects on glucose variation; specifically, our results are not sensitive to many confounders that one might imagine. *Second*, we wanted to how the robustness (i.e., stability of the computation) of our time series analysis methodology on real EHR data, despite all the measurement complexities present. *Third*, we wanted a population that, upon considering higher order effects, would have the potential to be stratified into different types or categories. *Fourth*, because EHR data are extremely complicated, we did not want to build in any more *a priori* notions of signals than were absolutely necessary. And *fifth*, because EHR data are not carefully collected physiologic data, to interpret EHR-data-driven results we must understand what EHR-based biases exist, and we cannot observe unknown EHR-specific biases when we choose a carefully manicured data set. Or, written differently, one of the purposes of this paper is to demonstrate how to derive a signal that is not sensitive to the alignment of patients, noisiness of feeding schedules, and other EHR-specific randomness, yet conveys useful information for population.

Because we use a very broad population, it is important to comment on the complex nature of the composition of the EHR data as a data source. To do demonstrate this, as an example, consider the hypothesis that diabetics would have the most frequently *recorded* glucose values. A careful verification of this hypothesis is both a substantial research question, and is out of the scope of this work. Nevertheless, we can make a bit of an inference into the validity of such a statement. Among the 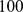 most measured patients in the CUMC EHR, 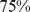 have at least one billing code for diabetes [13]. In contrast, if one considers a random set of patients, the proportion that have several glucose measurements within 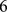 hours of one another who have at least one billing code for diabetes falls to 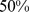 [13]. Meaning, within the general population of patients who are sampled at least as frequently as once every 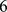 hours, at least half are *not* diabetic. One can imagine many plausible reasons for this; one example might be that a substantial portion of the glucose measurements come as part of a panel with other measurements in which case many of the glucose measurements would be measured as part of a routine for caring for particularly sick patients, such as patients admitted for congestive heart failure. Related issues regarding over or underrepresentation are difficult to address because of the relativity of the expected rates of measurement. In the United States, 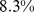 of the population (as of January 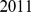) had diabetes, thus assuming a uniform measurement of glucose of US residents, diabetics are overrepresented in our data. It is unknown whether they are over or under *measured* relative to desired clinical protocols. Nevertheless, the data set we use contains a large number of both diabetic and non-diabetic patients who have frequent glucose measurements so features of intra- and inter-group dynamics are resolvable.

In addition to the random EHR population, we have included two relatively well measured patients from the CUMC EHR. We have included these patients to demonstrate that, despite potential population-aggregation effects on glucose variability (recall that Albers and Hripcsak [12] detailed how aggregation of different sources can affect a TDMI signal), the results we observe are present in well measured individuals too. This decreases the likelihood that our results are confounded by population aggregation alone. These patients were selected from among the 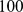 patients with the most glucose values in the CUMC EHR and they represent the two typical types of patients; the TDMI analysis of this subpopulation and others can be found in Albers *et al* [13] [25]. Both patients were sick, with different illnesses, and were hospitalized during some, but not most, of their measurements. Neither patients' glucose measurements come primarily from the ICU setting. Among this set of patients, there is not very much variation in the TDMI signal; we chose one patient (whose record is roughly 3 years long) with the weakest signal and one patient (whose record is longer than 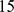 years) with a signal of average strength among this set of 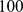 patients. Note that even the set of 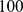 patients with the most glucose values is remarkably diverse when considering the notes for the patients. Some of the afflictions among this set of patients includes: pancreatic cancer, chronic kidney disease (CKD) (some CKD patients have type 1 or 2 diabetes and some do not), organ transplants of various types, type 1 or type 2 diabetes with various degrees of compliance with treatment, congestive heart failure, etc. Moreover, about 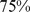 of these patients are presumed to be diabetic (either type 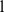 or 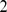). Due to the complexity of the models and patients, resolving the source of the higher order features of the TDMI distribution (e.g., the higher order moments) of the 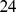-hour TDMI peaks among patients is beyond the scope of paper.

#### 0.4.2 Neural intensive care unit subpopulation

The NICU population is a much more narrow population, and because this population is acting as a control in some sense, it is important to detail their nutrition in a more detailed fashion. The entire set of 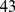 patients was administered enternal nutrition (i.e., via a feeding tube) starting within 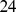 hours of aneurysmal repair [26]. While the enternal nutrition was continuous when given (denoted continuous feeding), there were random episodic gaps where nutrition was withheld (random Nil per os (NPO)). Specifically, the feeding is suspended before invasive procedures (e.g., surgery, extubation), when there are high gastric residuals (i.e., when there is a lot of residual food left in the stomach), when there exists intestinal obstruction (ileus), and when the patient has diarrhea or is aspirating the food. The existence of these random gaps in nutrition are the reason why one of the model populations is continuously fed with random feeding gaps. The enternal nutrition was the primary source of nutrition (less than 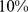 of the caloric intake came from other sources such as drugs). The primary target for each patient was 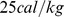 (or 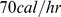) and the primary caloric source was Osmolite. The NICU population does receive insulin; how and why is complex and is discussed in detail in Schmidt *et al.* [27]. We do not attempt to control for insulin because it is difficult to foresee whether it matters; our results will show that the insulin regimen in the NICU population does not affect our results to first order in statistical moment (i.e., the mean). Finally, note that within the NICU population, less than 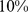 of the patients are diabetic; removing them does not alter the results.

#### 0.4.3 Contrasting the two patient populations

Conceptually, there are four important differences in these populations: **(i)** the EHR broad population is uncontrolled and monitored poorly (it is the general patient population after all) whereas the NICU population is highly controlled and monitored; **(ii)** the EHR broad population has an unknown and uncontrolled feeding pattern whereas the NICU population is being fed continuously and in a very controlled and documented fashion; **(iii)** the EHR broad population represents a diverse set of humans with diverse and unknown health states whereas the NICU population represents a very sick population whose degree of acuity is considerably higher and more narrowly defined than that of population one; and **(iv)** while the detailed understanding of metabolic function is unknown in both populations, it is very likely that the metabolic functioning of patients in the NICU population is substantially more compromised. Thus, the NICU population functions roughly as a *control to isolate the effects of continuous feeding on glucose daily variability* because this population has relatively few normal external physiological forcing mechanisms (e.g., sleep cycle, daily exercise, real mealtimes, etc.). In contrast, the broad EHR population is meant to represent the population at large whose feeding pattern is uncontrolled, highly discontinuous, and has unknown regularity.

### 0.5 Computational methods

We use two diagnostics for the EHR and model glucose time series, **(i)** intra-patient normalized glucose by hour, and **(ii)** the TDMI of the glucose time series (Albers and Hripcsak [13] explains how the TDMI can be applied to a population). It is important to note that the reason we chose the TDMI is that, when applied to a population, it affords the eventual possibility of stratifying patients by predictability (cf. conjecture one in Albers and Hripcsak [13]).

With respect to (i), we normalize each patient to mean zero and unit variance, and then calculate the mean and variance of glucose by hour over the population. We do this because there is a high degree of individual variability within each population, and individuals were measured differently from each other. Therefore, to resolve a property such as the by-hour daily variation of glucose values, we must remove inter-individual variation. Without this correction, inter-individual variation and therefore population aggregation effects became the first order effects. Nevertheless, we will show the normalized glucose variation for an individual patient to demonstrate that individuals mimic the population.

With respect to (ii), we calculate the TDMI [13] [28], [29], given by:
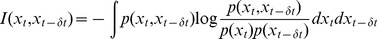
(13)where 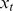 and 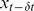 represent an ensemble of *all the intra-patient pairs of points in the population of time series separated by a time* 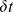 and 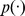 denotes the probability density function (PDF) of those ensembles; note that the TDMI captures linear and nonlinear correlations in time, which differs from, say, auto or linear correlation calculations (to see this applied to kidney function, see Albers and Hripcsak [12], and for general application, see Albers and Hripcsak [13]). Finally, to calculate the TDMI, one must estimate the joint and marginal PDFs, here we used a kernel density estimation (KDE) routine [30] implemented on MATLAB.

In general, the TDMI is a unit-less quantity; a TDMI of 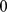 (within bias) implies that there is no correlation between sequential values in a time series for a given 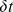. TDMI values begin to become important when they exceed the expected bias associated with calculating the mutual information, which is approximately 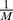 where 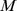 is the number of pairs of points used to estimate the TDMI (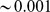 in this experiment). With a perfect correlation between sequential values, the TDMI will be equal to the entropy (or auto-information) of the series, which is numerically equal to the TDMI at 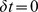 (and is calculated automatically as part of the experiment). In this experiment the entropy was about 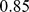 and represented the maximum TDMI. (In most of our experiments, the entropy is in the 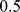 to 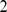 range.) Note that perfect correlation of a constant function (implying PDFs that are 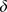 functions) yields a TDMI of zero for all 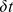.

With respect to the models, the ODEs were integrated over time-periods ranging from seven days to three weeks. A standard fourth-order Runga-Kutta integration routine, with a step-size of 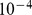, was utilized.

## Results

### 0.6 Basic physiological synopsis

**Figure 1:**
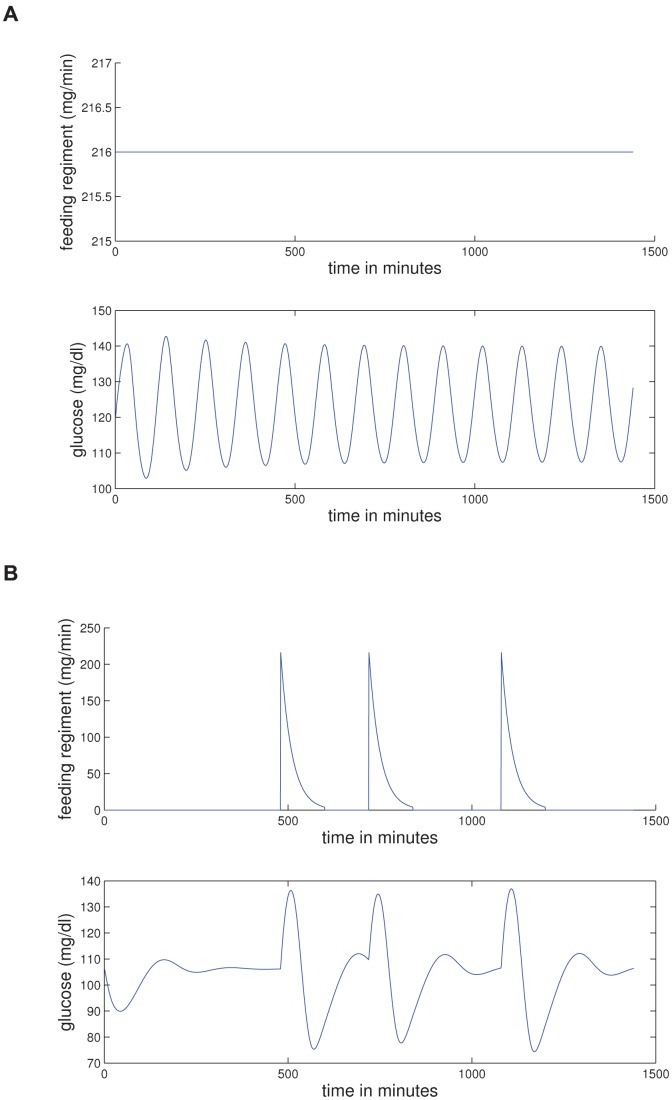
Depicted above are (a) the glucose for the standard glucose-insulin model with continuous feeding; and (b) the glucose for the standard glucose-insulin model with realistic meal structure. (a) Glucose-insulin model with continuous feeding and glucose response. (b) Glucose-insulin model with three meals and glucose response.

Figure 1 details the feeding-glucose response for the models. The point of this figure is to depict the basic building blocks that will be aggregated into a population. Figure 1(a) demonstrates that, relative to the model, a *continuous* infusion of glucose induces a periodic oscillation in intravascular glucose whose period is on the order of minutes; note that verification of this signal in humans can be found in Fig. 1 of Sturis *et al.* [19] or more generally in Lang *et al.* [31]. Furthermore, note that *in this case* the glucose oscillation is *exactly symmetric about its mean*, implying that long term averages of the glucose-insulin response should be a constant — this fits with the intuitive control theory vision of the glucose-insulin cycle. Figure 1(b) illustrates the glucose oscillation structure that is induced when the feeding pattern consists of three realistic meals given at 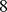, 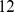, and 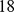 hundred hours respectively. Note that the peaks and length of time over which the glucose response exists depends on the magnitude of the calories in the meal — one way of conceptualizing this system is as a forced oscillator with damping that depends on caloric input and metabolism. Also note that the when caloric intake is a pulse, the glucose-insulin response is *not exactly* symmetric about the mean or baseline. In particular, isolating the glucose response and integrating the response relative to the baseline yields a very small but negative number, meaning that the overall glucose level is depressed when integrated over the course of the meal and response relative to this model.

### 0.7 Diurnal variability of glucose in a population

**Figure 2:**
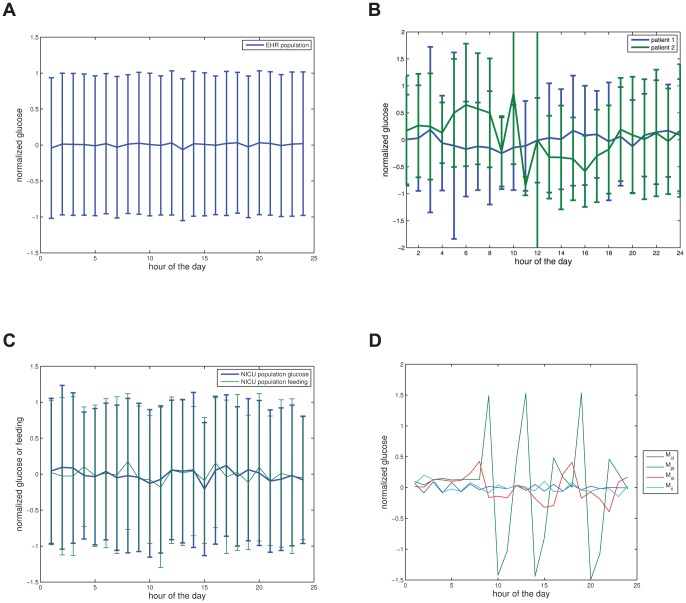
Depicted above are (a) the mean and standard deviation in glucose, by hour, for 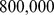 patients whom have been normalized to mean zero and variance one, with at least two glucose measurements from the CUMC EHR; (b) the two individual patients mean and standard deviation in glucose measurements by hour, note the variability in patient 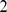 for which there are far fewer measurements than for patient 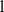; (c) the mean and standard deviation in glucose and *enteral* (i.e., tube) feeding rates, by hour, for 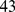 normalized patients in the neural ICU; (d) glucose, by hour, for various different model feeding patterns. (a) Normalized population glucose by hour. (b) Single patient normalized glucose by hour. (c) Normalized NICU population glucose and feeding by hour. (d) Normalized model glucose by hour.

With the basic building blocks of glucose-insulin response in place, next consider Fig. 2 which details the hourly glucose variability within the data sets and models. In particular, in Fig. 2(a) the hourly glucose variability for the EHR population displays *no observable diurnal variability* or signal. While we expected the short-term oscillations to average out we also expected to observe a small but statistically significant signal on a 24-hour cycle that matched meal times. More specifically, we expected a small diurnal signal because: **(i)** humans eat periodically, which, intuitively, implies that glucose would be broadly higher over meal times; and **(ii)**, there exists a *weak but present diurnal variability* in kidney function that was observed on the *same data set* [12] — which was surprising in and of itself because kidney function is not normally believed to have a strong diurnal signal.

Before we give a more technical explanation as to why we, equipped with the constructive model, would not expect to see any diurnal variation in raw glucose values when averaged over a population, it is important to compare the data-based signals in Figs. 2(a) and 2(b) with the modeling results shown in Fig. 2(d) to draw a few observations/conclusions. *First*, constant feeding in the model for a population leads to constant (averaged by hour) glucose which agrees with the data-based result (NICU patients) of Fig. 2(b), and thus verifies that relative to hourly glucose variability, the model correctly predicts the observations. Of course, because many feeding patterns produce the same signal in hourly glucose variability, this does little to verify that the model represents the endocrine system faithfully — to achieve this we need a different, less ambiguous data-driven signal. *Second*, the periodically driven individual has the expected daily meal response structure; but the signal is too clean to realistically represent an individual or a population because the meals are given at exactly the same time every day. *Third*, the random feeding produces no diurnal signal and thus agrees with the data-based result (random population) from Fig. 2(a), meaning that it is possible either that the model does not depend on strongly on feeding structure or that the by-hour glucose is not good enough to detect feeding structure and differentiate the respective populations. In the section that follows, we demonstrate that the second statement is the correct conclusion. *Fourth*, the noisy periodic case has wide, weak diurnal peaks at meal times, which differs from what is observed in the data; however, the primary reason the diurnal structure in daily glucose variability is retained in the models with noisy periodic-like feeding is that the meals are uniformly distributed within two hour *disjoint* intervals. We know from further experiments that increasing the *diversity of the location of the mealtime windows between individuals, while retaining the noisy mealtime structure within individuals*, allows the model results to reproduce the population signal shown in Fig. 2(a) more faithfully. And *fifth*, considering the model output shown in Fig. 1(b) where the glucose-insulin response to a meal *is roughly symmetric about the baseline glucose level*.

Armed with the above information, we can now make a more technical argument as to why there is no signal in the hourly variation of glucose. The simplest explanation for the lack of observed diurnal signal in hourly glucose values relies on four observations, **(i)** we are aggregating/averaging many sparsely measured sine-wave-like signals whose periods are much shorter than an hour (that they come from many or a single patient is largely irrelevant), **(ii)** these averaged waves have random starting times, **(iii)** the averaged waves have different periods (e.g., because patients are diverse), and **(iv)** the averaged waves have are symmetric about their means (which are normalized to zero). Such signals, when averaged, will yield a constant function. To see why this is the case, consider a collection of sine waves that have different periods whose *average converges* to something finite; the sum of those sine waves will converge to 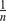, where 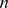 is the number of sine waves being averaged. This does not mean that there isn't a diurnal dependence within glucose (in fact, we find there is using a derived value), or the glucose/insulin response following a *specific meal* isn't observable within EHR data, because it is. But, when one averages over time, even for an individual patient who is not being tube-fed (cf. Fig. 2(b)), variation in the daily average glucose is not observable because of the noisy meal schedules (which affect phases, periods and amplitudes), the act of averaging, the structure of the glucose/insulin response to food (the response is order minutes not hours), and the course resolution of measurement. One can imagine more complicated reasons for why there is no signal in Figs. 2(a) and 2(b), but the simple answer without complicating factors (e.g., diabetes, NPO, acuity) — that aggregation/averaging plus the dynamic type obliterates any signal — is enough to remove the signal in the EHR population, individuals, and all the models. Thus, these other complications, while acting as possible contributors to the lack of signal, are neither necessary to remove the signal, nor observable given only the raw glucose values.

### 0.8 Diurnal variability in nonlinear correlation of glucose

**Figure 3:**
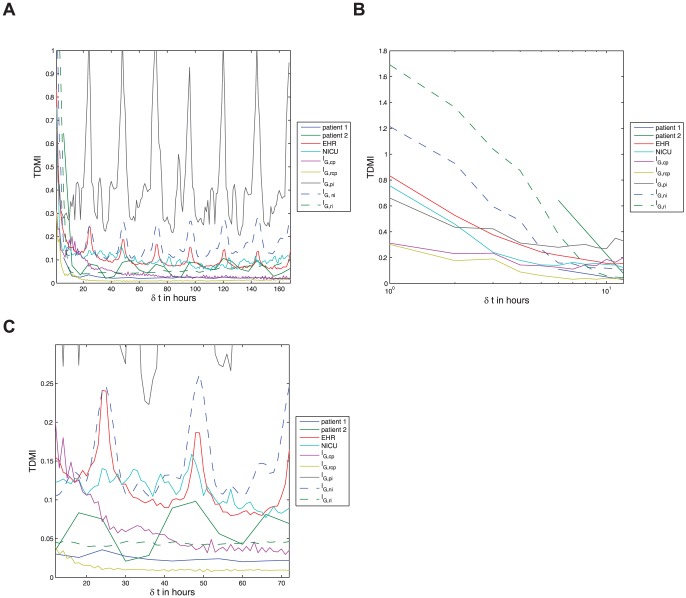
Depicted above are (a) the TDMI curves for all EHR-data based populations and model output for all feeding patterns resolved to one hour intervals for time delays of up to one week, note the sharp decay in TDMI in all cases, and the diurnal peaks in all periodically fed populations or models — *note* this plot is split into dynamical regimes in Figs. 2 and 2; (b) the TDMI curves for all populations and models over time-delays of 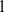 to 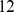 hours; and (c) the TDMI curves for all populations and models from 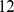 to 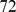 hours, notice the diurnal peaks in all periodically fed populations or models. Recall that the model feeding patterns are given by: 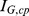 — continuously fed population; 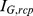 — continuously fed population with random 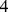 hour gaps; 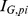 — periodically fed individual; 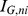 — noisy-periodically fed individual; and 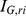 — a randomly fed individual. (a) All data sets and models — a global view of the TDMI. (b) All data sets and models — feeding scale TDMI for 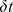 of 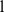 to 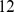 hours. (c) All data sets and models — diurnal scale TDMI for 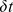 of 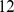 to 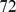 hours.

Finally we arrive at the nonlinear-correlation variability in glucose as quantified by the TDMI. Figure 3(a) frames the TDMI over an entire seven day time-delay window and can, in a sense, be split into two dynamical regimes, the TDMI for 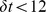 hrs and for 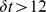 hrs. To highlight this difference, and to aid readability, Figs. 3(b) and 3(c) are Fig. 3(a) split at 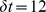 hrs. As previously stated in 0.5, it is possible to use the *distribution* of the TDMI to stratify the population at a given 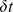; here we will refrain from analyzing these higher order (relative to the distribution moment) effects and instead concentrate on the first order effects as defined by the *mean* TDMI values that are shown in Figs. 3(a)–3(c). To be clear, note that Fig. 3 contains the TDMI signals from *both* EHR data (the random EHR population, the two individual patients, and the NICU population) and model output (there is a TDMI signal corresponding to each of the feeding patterns introduced in section 0.3). Thus, we are explicitly comparing the TDMI signals of the EHR data sets against themselves as well as the TDMI signals of the model output. With this in mind, the following features of Figs. 2-2 are of note: **(i)** all models and data sets show a sharp decay in TDMI between one and twelve hours; **(ii)** one of the individual patients has weak diurnal peaks in the TDMI at 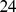 and 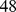 hours while the other patient has diurnal peaks for several days; **(iii)** the NICU population shows no long term structure in the TDMI, although there does remain a constant amount of TDMI present; **(iv)** the uncontrolled EHR population shows diurnal peaks in the TDMI, and the magnitude of these peaks decays with time; **(v)** the continuously fed population model, after the decay within twelve hours, shows a weak hump at eighteen hours that is a function of the exact symmetry of the periodic oscillations in glucose, followed by a decay to small, constant, TDMI — *thus, this model case accurately represents the NICU patients*; **(vi)** the periodic *individual* model patient without noise has a good deal of TDMI as well as sharp diurnal peaks and — note that from this it is self-evident that an individual patient with a continuous feeding regimen would also have a high level of TDMI, albeit without the sharp 24-hour peaks; **(vii)** noisy periodic model has, after the sharp decay at twelve hours, diurnal peaks in the TDMI with *non-decaying* magnitude — *thus, this model mostly closely represents the real EHR population, and in fact the two overlay up to about* 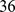 *hours*; **(viii)** the TDMI for the randomly fed model case has no long term structure — *thus, the TDMI helps distinguish the constant feeding, the random feeding, and the noisy periodic feeding models*. To consider more detailed analysis, it is instructive to split Fig. 2 into two regimes, 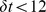 hrs, and 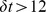 hours.

The most important feature of Fig. 2, which shows the TDMI for 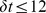 hrs, is that the collection of TDMI curves are *bounded from above by the random feeding and below by the population with continuous feeding models* respectively. The random meal case has the most TDMI within the first 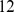 hours because the random feeding case maximizes the amount of observed TDMI per mealtime period. This maximization occurs for two reasons: **(i)** isolated meals have a large amount of TDMI that persists over approximately four hours; and **(ii)**, meals are uniformly distributed over the 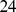 hour period and are unlikely to overlap. Said simply, the TDMI for the random meals population with 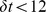 largely represents the pure *intra-meal* TDMI, which is the maximum TDMI amongst the models (and apparently real populations) we examine. This argument is further backed-up by the fact that the randomly fed population has the sharpest decay in TDMI. The reason why the TDMI for the population of continuously fed patient model is a lower-bound is due to a combination of aggregation effects and superpositions of periodic orbits. To understand this, recall Fig. 1(a) and note that each member of the population of continuously fed patients will have orbits with *different amplitudes and frequencies* and that aggregating them together at a given 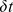 will produce a distribution that will closely resemble a uniform distribution — the distribution that minimizes TDMI over all distributions. All the other cases fit in between these two extreme situations.

The longer the time (separation) scale is shown in Fig. 3(c) and includes the TDMI for all cases over time-separations of 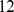 to 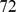 hours. Begin by noting that there is no structure in TDMI signal for the NICU population as well as the random feeding and continuously fed population models. Thus, using only the TDMI and the normalized hourly glucose, it is difficult to distinguish the continuously fed population from the randomly fed population. In contrast, the EHR population, by displaying the diurnal peaks, is easily distinguishable from the NICU population; thus the TDMI helps distinguish the EHR and NICU populations in a way that analysis of the raw glucose values could not. Moreover, because the noisy feeding and EHR populations strongly resemble one another (they are nearly identical for 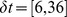 hours), and because the exactly periodic feeding yields far to much TDMI, *the difference between the EHR population and the NICU population is likely due to noisy, but specifically structured (i.e., not totally random) meal times*. This conclusion both confirms that EHR data reproduces what is believed to be the first order glucose dynamical effect, nutrition, and verifies that the ultradian model can represent humans for much longer time scales than hours to minutes. Finally, even the individual patient with the weakest signal shows a peak at 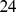 hours and a weak peak at 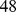 hours, which is consistent with the EHR-based TDMI signal.

### 0.9 Resultant synopsis

Based on Fig. 3(c), *the most basic and elemental result is thus: the model output can be used (in conjunction with the TDMI) to correctly predict the distinction between general EHR patients and NICU patients on time scales longer than a day*. Moreover, the observed TDMI signal for the EHR population represents *noisy, but structured meal times over the population*; meaning, *we can detect human behavior patterns in EHR data and test them against physiological models*. That is, adjusting the feeding in the model alone was enough to account for the difference in the observed TDMI signals and thus to distinguish the populations to first order in statistical moment (i.e., mean TDMI), all without injecting difference (e.g., differences in mean age) into the parameters. This implies that EHR data can at least resolve some first order physiological effects. At a finer resolution, while the first order moment of the TDMI (i.e., predictability) can be used to separate the two populations of patients because of how nutrition is ingested, understanding the second order moment (i.e., the variance of the TDMI peaks for a fixed 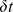) is more complicated and is beyond the scope of this paper. More explicitly, it is likely that the higher order moments of the TDMI peaks will depend, to some unknown level of detail, on the health state of the patient. Moreover, because even narrow EHR populations are relatively diverse and as yet unquantified in the context at hand, and because even the simple model we used has about 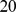 parameters that we hold fixed *for all populations examined here* that are nevertheless are available for variation, resolution of the higher order moments of the TDMI peak is beyond the scope of the current paper. Nevertheless, preliminary analysis seems to point to the TDMI being monotonically dependent on nutrition and the functioning (or artificial regulation) of the pancreas. We were able to use EHR data to test a physiological model for a population, but, as is the case with many other data-driven fields, derived values (i.e., the TDMI) were more helpful than the raw values. Finally, the relatively simple constructive glucose/insulin model *can accurately represent a population over much longer time periods than it was designed to represent*. Or, more specifically, while the model we use here is an ultradian model designed to be applicable on a time-scale of much less than a day, the model nevertheless appears to be applicable over time periods considerably longer than a day.

## Discussion

### 0.10 Summary discussion

The end goal of population physiology is twofold: **(a)** we want to derive population-scale, data-based signals over medium to long time-scales in a way that can be connected to constructive, mechanistic models to further the understanding of human physiology; and **(b)** we want to be able to use these verified, constructive, mechanistic models to affect the health of human beings via clinical care. In this paper, we have demonstrated (a) but not (b), primarily because glucose/insulin modeling is not yet at a stage were it can be applied to affect clinical care in a direct manner. Nevertheless, we have begun one of the necessary steps for implementing (b): we have demonstrated that a mechanistic model of endocrine dynamics can accurately represent humans over the longer time scales that are relevant to clinical outcomes.

Scientifically, the results in this work demonstrate and imply that: **(i)** the output from a simple glucose/insulin model can be used to predict the difference between EHR and NICU patients over time periods longer than a day; **(ii)** glucose measurements for a population yield diurnal variation in *correlation*, but glucose dynamics behave in a way (i.e., oscillations about a mean whose period is order minutes) such that *diurnal variation in raw glucose values* is difficult to observe; **(iii)** “self-fed” humans *do* have a diurnal TDMI signal in glucose; **(iv)** “normal” human glucose values *do* display an initial decay in correlations (between subsequent measurements) to a relative baseline within 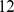 hours; **(v)** the models with the noisy but structured meal times match the diurnal TDMI EHR signal, thus the diurnal cycle in predictability of glucose is primarily driven by nutrition (not an internal clock); **(vi)** EHR data can resolve a signal that spans multiple time scales and can be used to test physiological models; **(vii)** that the standard glucose/insulin model [19] is applicable beyond the time-spans it was designed for; **(viii)** the NICU population and continuous feeding model TDMI signals match one another — in particular, humans being fed continuously *do not* have a diurnal TDMI signal or any structured signal at all; and **(ix)** EHR data resolves human social behavior — a meal time structure influencing glucose physiology.

### 0.11 Potential impacts of integrating EHR data with mechanistic models

There are two broad avenues through which the the integration of mechanistic, constructive models with EHR-data can help advance clinical care, data assimilation (and control theory) and *in silico* experimentation.

*First*, given a mechanistic model, incorporating data into the model to forecast the future is done using data assimilation [32] [33]. To control the system all that is needed is an addition (the controller) that codifies a desired outcome and a means of achieving it relative to the parameters that are available for adjustment. Data assimilation has not been used in this context, control theory has a limited history in biomedicine but is emerging as an important technique in a clinical context.

*Data assimilation* (DA) (e.g., a Kalman filter), combines *observed* data from the current (and often the past) state(s) of the system with underlying dynamical principles governing the system (i.e., a constructive model) to make an accurate estimate or forecast of the true state of the system at any given time, *including variables that were not measured*. The DA prediction is referred to as an *analysis*. This *analysis* output is fed back into the model to make a prediction or forecast about future state of the system. Therefore, from a more practical standpoint, DA schemes perform two functions: **(i)** they reconstruct the state variables of a model, including both observed and unobserved variables; and **(ii)**, they forecast the future in a way that can be directly tested with future measurements (and used to implement control theory). Thus DA schemes are the explicit way that data are injected into constructive models such that predictions and forecasts can be made. This allows for “patient forecasts, ” where different outcomes can be based on current and future observations and/or hypothetical data, thus allowing for exploration of “what if” scenarios with patients. This in turn allows us to take a more personalized view of treatments for patients in clinical applications. Finally, some DA schemes (e.g., unscented Kalman filters) allow for “empirical observability,” or the ability to *rank* which variables are the most useful for reconstructing the other variables, thus allowing us to determine the most useful clinical variables, in some sense. Sedigh-Sarvestan *et al* [38] applies a DA applied to the model in this paper that includes empirical observability ranking of parameters and variables.

*Control theory* [33] [34] [21] [35] applied to solve biomedical and clinical problems has a very successful but limited history. Recall that traditionally control theory has been used in engineering in diverse contexts ranging from cruise control in a car to stabilizing and flying jet aircraft to optimizing manufacturing processes. Examples in biomedical contexts include implantable cardioverter-defibrillator or pacemakers to cope with irregular heartbeats, work toward creating an artificial pancreas [36], and to design treatments for prostate cancer [37]. To apply (optimal) control theory to any problem, one usually requires three components, an explicit model of the process to be controlled (e.g., the glucose/insulin model shown here), a statement regarding the constraints of the system (e.g., fixed or disallowed parameter settings, initial conditions, boundary conditions, etc.), and specification of the performance (e.g., how tightly one wants to control glucose) [34]. EHR data will likely be the only data available on a population scale that can be used to test a models, specify the constraints, and specify the desired performance (based on retrospective EHR-data based study) based on desired outcomes. With a control theory infrastructure in place for a given physiologic system applications are very broad. For instance, one could design a controller to regulate glucose in an ICU setting (cf. Sedigh-Sarvestan *et al* [38] where the an unscented Kalman filter is applied to the model in this paper), one could use the controller to design optimal treatment strategies over long periods of time for outpatient diabetics, or one could design artificial organs such as the artificial pancreas project [39] [40] [41]. But these possibilities are only possible in practice when we have a constructive model available.

*Second*, if a constructive model is good enough, and can be verified well enough, it can be used to test new drugs and treatments even without data (e.g., outside of a personalized medicine approach where data assimilation is used). Such a situation is referred to as *in silico* experimentation, and it has already begun in some contexts. For example, recently an endocrine model of the type 1 diabetes, being used in the context of developing an artificial pancreas [36], has been approved by the FDA as a substitute for animal trials for preclinical trials [41] [39] [40]. In this case, artificial data are created (based on real data, but not a DA analysis), and then different treatment strategies are tested. This approach has the potential to greatly accelerate the rate of advancement of therapy in many different contexts.

### 0.12 Looking forward

Looking forward, population physiology suffers from the lack of existent, time-dependent signals; discovering such signals that can be related to physiological models is where many current opens problems lie. Said differently, before one can go about refining models and understanding dynamics mechanistically and over longer time periods, one needs actual data-based signals, or stylized facts [42], that can suggest and motivate refinements in the models via *testing* of those models before DA or control theory can be applied. Moreover, we need to approach defining populations by their dynamics from two directions, stratifying populations by known characteristics (e.g., presence of type 1 or type 2 diabetes) and observing signals and constructing signals, and using those signals to stratify populations.

To drive mechanistic physiologic modeling forward, and to make it more useful, a practical, EHR data-integrated approach that allows for either interaction with clinical care or better reflection of known physiological problems is necessary — for it is through qualitative understanding of models as dynamical and control systems [43] that actionable clinical interventions will come. Relative to glucose/insulin regulation, in some circumstances, monitoring and correcting for hyperglyceimia can help reduce mortality significantly [44] (note, the issue of how tightly to control glucose in the ICU is complicated and controversial). Nevertheless, correlation is not causation; the *mechanistic reasons* why glucose control in ICU populations helps with outcomes is not well understood, and thus optimal clinical interventions remain unavailable (cf., the introduction in Moghissi *et al.* [45]). The inevitable conclusion is that glucose/insulin dynamics and time implications of those dynamics are poorly understood on longer time scales. Moreover, the current state of glucose/insulin physiological modeling does not have a mechanism for understanding the fundamental physiological problems (i.e., longer term effects of glucose dynamics) that can suggest productive clinical interventions (e.g., ICU glucose control and regulation). But, again, such models cannot be developed without impetus, and that impetus must come in the form of concrete, data-based signals. While the data scarcity has made such signals difficult to come by, EHR data will put the data scarcity problems behind us and replace these problems with new signal processing problems that must be overcome. This paper represents a step forward in this direction by using EHR data to discover a physiologic-based signal that is connected to physiologic-based models even in the circumstance where direct observation of the physiological variable does not yield a signal that can stratify the population.
